# Elective fertility preservation before and after the Covid-19 pandemic outbreak

**DOI:** 10.5935/1518-0557.20240078

**Published:** 2025

**Authors:** Nivin Samara, Tal Israeli, Tal Shahar, Sagi Levi, Shimi Barda, Asnat Groutz, Foad Azem, Hadar Amir

**Affiliations:** 1 Racine IVF Unit, Fertility Institute, Lis Maternity Hospital, Tel Aviv Sourasky Medical Center, Tel Aviv, Israel affiliated to the Sackler Faculty of Medicine, Tel Aviv University, Tel Aviv, Israel; 2 Sami Shamoon College of Engineering, Ashdod, Israel

**Keywords:** Covid-19, elective fertility preservation, outcomes, ovarian stimulation, pandemic

## Abstract

**Objective:**

To compare the number and outcomes of elective fertility preservation (FP) before and after the Covid-19 outbreak.

**Methods:**

This retrospective study of 574 women who underwent elective FP between 01/2017-12/2021 included 123 women who underwent the procedure before and 451 who underwent it after the Covid-19 outbreak. The change in the number of women who underwent the procedure each month before and after the pandemic was calculated. The ovarian stimulation outcomes were compared between the two groups.

**Results:**

The post-Covid-19 group included significantly more single women compared to the pre-Covid-19 group (93.8% *vs*. 91.1%, *p* = 0.024). A progestin-primed ovarian stimulation protocol was followed only among the women in the post-Covid-19 group (18.8% *vs*. 0%, *p*<0.001), and their gonadotropin dose was significantly lower than that of the women in the pre-Covid-19 group (3164.6±842.87 mIU/mL *vs.* 3426.5±1080.63 mIU/mL, *p*=0.014). There were no significant group differences in ovarian stimulation duration (*p*=0.069), peak estradiol level (*p*=0.606), number of retrieved and mature oocytes (p=0.545 and *p*=0.364, respectively), oocyte maturity rate (*p*=0.719) or the number of women who cryopreserved embryos (*p*=0.861). High levels of basal FSH and low antral follicle counts correlated negatively with the total numbers of retrieved and mature oocytes.

**Conclusions:**

A rapid and sustained increase in elective FP after the outbreak of the Covid-19 pandemic that significantly surpassed pre-pandemic levels was observed. There was no significant difference in FP outcomes between the two time periods.

## INTRODUCTION

Coronavirus disease 2019 (Covid-19), caused by the SARS-CoV-2 virus, was declared a pandemic by the World Health Organization (WHO, 2020) on March 11, 2020. The first Covid-19 case was confirmed in Israel on February 21, 2020. As the number of reported cases increased worldwide, the Israeli government announced a strict lockdown on March 19, 2020 in an attempt to prevent the further spread of the disease (Rossman *et al*., 2020). At that time, in accordance with the recommendations of the American Society for Reproductive Medicine and the European Society of Human Reproduction and Embryology (ASRM, 2020; ESHRE, 2020), all fertility services in Israel with the exception of clinically urgent (cancer-related) gamete/embryo cryopreservation were suspended for one month and then gradually resumed.

A woman’s reproductive window is narrow. After the mid-thirties, their fertility potential decreases gradually, lowering after 35 years of age (Dunson *et al*., 2004; Sozou & Hartshorne, 2012). Their fertility continues to decline every year until menopause because the number and quality of the primordial follicles of oocytes decrease, a process associated with lower chances of the oocytes being fertilized, and higher risks of abnormal embryos and fetal loss (Hook, 1981; Faddy *et al*., 1992; Nybo Andersen *et al*., 2000). In Israel, as in other Western societies (Mills *et al*., 2011; Schmidt *et al*., 2012), the proportion of women delaying childbearing until the late 3rd-early 4th decade of life has greatly increased. Elective oocyte/embryo cryopreservation bestows upon women the opportunity to conceive their genetic offspring in the future (Cobo *et al*., 2013). The rates of survival of oocytes, pregnancy, and cumulative live births are highly dependent upon the woman’s age at the time of vitrification and the number of oocytes in storage. The probability of childbearing is significantly lowered over the age of 35 years (Cobo *et al*., 2016).

The decision to carry out elective fertility preservation (FP) is influenced by many factors, including the woman’s age, lack of a stable partner, financial considerations, self-realization, the desire for a genetically related child, education, employment, and career statuses (Hodes-Wertz *et al*., 2013, Nasab *et al*., 2020). The Covid-19 pandemic has profoundly impacted the lives of many people through changes in the same above-mentioned factors, and it may therefore influence the decision-making process in the setting of elective FP as well.

Data on the impact of a Covid-19 pandemic on assisted reproductive technology (ART) are limited. Two studies have dealt with the effect of the pandemic on ART service utilization rates (Requena *et al*., 2020; Zhou *et al*., 2021), and three focused upon the implications of the Covid-19 pandemic on clinically urgent FP (Requena *et al*., 2020; Trawick *et al*., 2022; Roux *et al*., 2022) but not on non-urgent planned FP. One recent study explored the impact of the Covid-19 pandemic on the perception of planned oocyte cryopreservation in the United States and found that 15% of the participants altered their likelihood of considering oocyte cryopreservation: 52.6% reported an increased likelihood, and 47.3% reported a decreased likelihood (Huttler *et al*., 2022).

Elective FP in Israel is permitted between the ages of 30-41 years. During the period in which the current research was carried out, each woman was permitted by law to undergo up to four fertility preservation cycles and freeze up to 20 mature oocytes. Costs for the procedure are currently not funded in Israel. The number of elective FP cycles increased almost fourfold between 2015-2019 (129 cycles *versus* 487 cycles) (Research and Information Center, Knesset of Israel, 2021). A report submitted to the Knesset (The Israeli Parliament) in February 2021 stated that the months following the outbreak of the Covid-19 pandemic witnessed a significant increase of between 3 and 4 times in women undergoing elective oocyte cryopreservation (Research and Information Center, Knesset of Israel, 2021, unpublished data).

Our study aimed to evaluate the repercussions of the first Covid-19 pandemic outbreak on the number and outcomes of elective FP.

## MATERIALS AND METHODS

### Ethical approval

This study was approved by the ethics committee (Helsinki) of the Tel Aviv Sourasky Medical Center. (#0806-21-TLV). Informed consent was waived for this retrospective and anonymous analysis.

### Study population and participant recruitment

This retrospective study was performed between January 2017 and December 2021 at the IVF Unit, Fertility Institute, Tel Aviv Sourasky Medical Center, a tertiary university-affiliated medical center. Five hundred seventy-four women who underwent their first elective FP treatment were included, of whom 123 women underwent the process before Covid-19 pandemic (January 2017 to February 2020) and 451 women after the Covid-19 outbreak (May 2020 to December 2021). No elective FP were performed during the lockdown (March to April 2020). Only the first treatment cycle of each woman was included in this study.

### Data collection

All relevant data were collected from the computerized database of the hospital. The data in the electronic charts included the following: clinical details (age, body mass index [BMI], marital status, previous children, thyroid-stimulating hormone [TSH] levels, and prolactin levels) and fertility potential details (basal follicle-stimulating hormone [FSH] and antral follicle count [AFC]), ovarian stimulation details (protocol type, ovarian stimulation duration, total FSH dose, peak serum estradiol [E2] level, peak serum progesterone level, and type of final maturation trigger), and outcomes (number of retrieved oocytes, number of metaphase II [MII] oocytes and maturation rate [derived from the number of MII oocytes/number of oocytes aspirated]). Data on the number of women who cryopreserved embryos and the origin of the sperm were also collected.

### Ovarian stimulation, fertilization, embryo culture, and embryo transfer

Controlled ovarian stimulation was carried out by the gonadotropin-releasing hormone (GnRH) antagonist, progestin-primed ovarian stimulation (PPOS), or short GnRH agonist protocols. Ovulation was triggered with 0.2 mg of triptorelin (Decapeptyl; Ferring Pharmaceuticals) or 250 mcg of choriogonadotropin α (Ovitrelle; Serono, Geneva, Switzerland), or by a combination of both when at least three follicles achieved a diameter of 18 mm. Ovum pickup was performed 36 hours later, and the embryologists determined the total number of oocytes retrieved and the MII oocytes per cycle. All MII oocytes were cryopreserved. Conventional in vitro fertilization (IVF) or intracytoplasmic sperm injection (ICSI) was performed if embryo cryopreservation was planned. All of the embryos were incubated in the integrated EmbryoScope^TM^ time-lapse monitoring system (EmbryoScope^TM^; UnisenseFertiliTech A/S, Aarhus, Denmark, Vitrolyfe) from the time of fertilization until freezing.

### Statistical analysis

Data were analyzed with SPSS, version 25.0 (SPSS, Inc., Chicago, IL, USA). The data were summarized as mean±SD or number of responders (percentage) according to the variables. Significance was tested with the t-test, Mann-Whitney U test, χ^2^ test, and Fisher’s exact test as appropriate. A multivariate linear regression analysis was performed to control for age, basal FSH level, AFC, and protocol type as confounders for the number of retrieved oocytes, number of MII oocytes, and oocyte maturity rate. A *p* value of < 0.05 was considered significant.

## RESULTS

### Prevalence of FP before and after the Covid-19 pandemic outbreak

**Figure 1:**
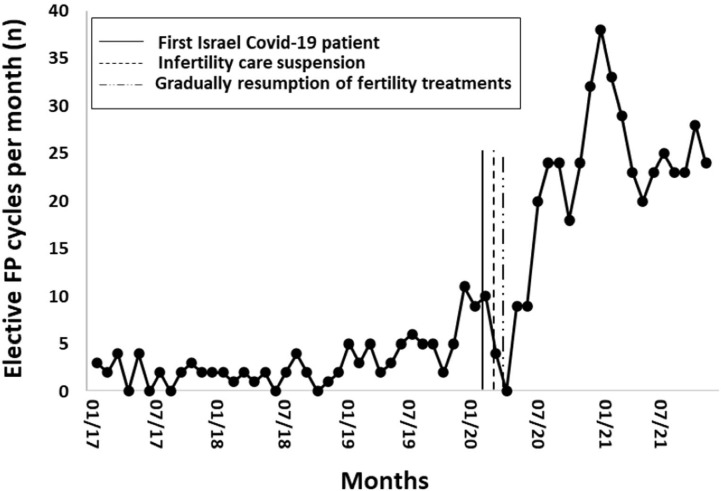
Number of elective fertility preservation (FP) procedures per month between January 2017 and December 2021.

**Figure 2:**
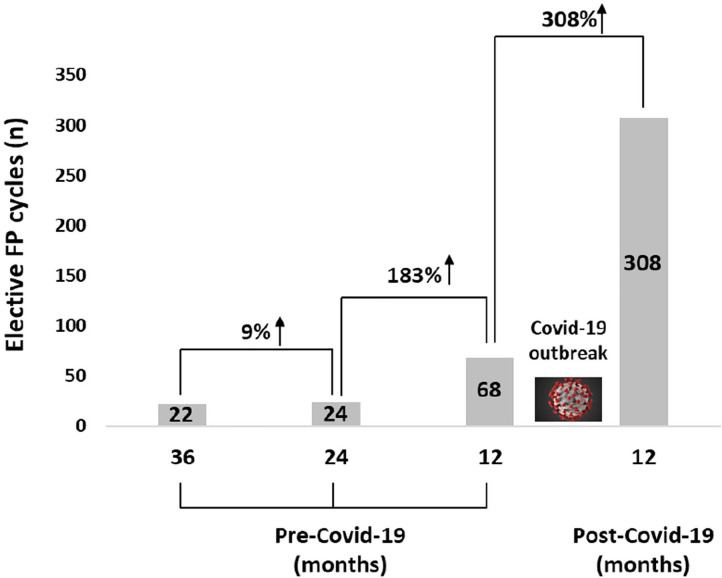
Number of elective fertility preservation (FP) procedures at 36, 24, and 12 months before and 12 months after the outbreak of the Covid-19 pandemic.

A total of 574 women who underwent elective FP were included in this analysis, of whom 123 underwent the procedure before pandemic outbreak and 451 after it. A consistently low number of women underwent elective FP until one year before the pandemic outbreak (01.2017-01.2019) when an upward trend began in 2019 and continued until the pandemic outbreak. Then, elective FP rates sharply decreased during suspension of the service (03.2020-04-2020), followed by a significantly steady increase (Figure 1). Three times more elective FP cycles were completed during 2019 compared to the previous years (an increase of 183%), and 4.5 times more cycles were performed during the 12 months following the first Covid-19 outbreak compared to the previous year (an increase of 353%) (Figure 2).

### Clinical characteristics of the study participants

The clinical characterizations of the entire cohort are detailed in Table 1. The BMI of the women in the pre-Covid-19 group was significantly lower than the women in the post-Covid-19 group (22.6±4.13 kg/m^2^ *vs*. 23.8±4.68 kg/m^2^, respectively *p=*0.028), however, the BMIs of both groups were within the normal range. Significantly more women were single in the post-Covid-19 group compared to the pre-Covid-19 group (93.8% *vs*. 91.1%, *p=*0.024). There were no other clinical differences between the two groups, including the women’s age (*p=*0.217) and the ovarian reserve markers FSH (*p=*0.989) and AFC (*p=*0.218).

**Table 1:** Comparison of clinical parameters between women who underwent elective fertility preservation before and after the outbreak of the Covid-19 pandemic.

Characteristic	Before Covid-19 (n=123)	After Covid-19 (n=451)	*p* value
Age (y)	35.8 (1.98)	35.6 (2.28)	0.217
BMI (kg/m^2^)	22.6 (4.13)	23.8 (4.68)	0.028
Marital status Single Married Divorced	112 (91.1%) 6 (4.9%) 5 (4.1%)	423 (93.8%) 5 (1.1%) 23 (5.1%)	0.024
Previous children No Yes	120 (97.6%) 3 (2.4%)	445 (98.7%) 6 (1.3%)	0.412
Day 3 FSH (mIU/mL)	8.5 (3.13)	8.5 (3.48)	0.989^^*^^
TSH (µIU/mL)	2 (1.42)	2 (1.06)	0.811^^*^^
Prolactin (mIU/L)	263.8 (123.91)	277.3 (166.76)	0.690^^*^^
AFC (n)	12.7 (7.01)	13.1 (6.22)	0.218^^*^^

Values are presented as mean (standard deviation) or number (%). A *p* value of < 0.05 was considered significant.

* The *p* value was calculated after log transformation for normal distribution.

BMI, body mass index; FSH, follicle-stimulating hormone; TSH, thyroid-stimulating hormone; AFC, antral follicle count; SD, standard deviation.

Note: Standard reference ranges: FSH: 1-9.2 mIU/mL; TSH: 0.5-4.8 µIU/mL; Prolactin: 108.78-557.13 mIU/L

### Ovarian stimulation data and outcomes

The ovarian stimulation data and outcomes of the two groups are summarized in Table 2. The use of the PPOS protocol was significantly higher after the pandemic outbreak compared to before (0 *vs*. 18.8%, *p<*0.001). No significant differences between the preand post-Covid-19 groups were found in the duration of gonadotrophin treatment (*p*=0.069), the peak E2 level (*p*=0.606), the peak progesterone level (*p*=0.575), the type of final maturation trigger (*p*=0.091), the numbers of retrieved oocytes (*p*=0.545) and of MII oocytes (*p*=0.364), oocyte maturity rate (*P* = 0.719), the number of women who cryopreserved embryos (*p*=0.861) or the sperm origin (*p*=0.421). The amount of FSH used was significantly higher for the pre-Covid-19 women (3426.5±1080.63mIU/mL) compared to the post-Covid-19 women who underwent fertility treatment (3164.6±842.87mIU/mL; *p*=0.014).

**Table 2:** Comparison of ovarian stimulation data and outcomes between women who underwent elective fertility preservation before and after the outbreak of the Covid-19 pandemic.

Characteristic	Before Covid-19 (n=123)	After Covid-19 (n=451)	p value
Protocol type GnRH antagonist Short GnRH agonist PPOS	121 (98.4%) 2 (1.6%) 0	357 (79.2%) 9 (2%) 85 (18.8%)	< 0.001
Ovarian stimulation duration (days)	10.9 (1.67)	11.2 (1.67)	0.069
GT total dose (mIU/mL)	3426.5 (1080.63)	3164.6 (842.87)	0.014
Peak E2 (pg/mL)	2680.2 (2101.53)	2752.2 (2322.03)	0.606^*^
Peak P (ng/mL)	1.3 (1.02)	1.1 (0.63)	0.575^*^
Final maturation trigger GnRH agonist hCG hCG+GnRH agonist	110 (89.4%) 12 (9.8%) 1 (0.8%)	424 (94%) 21 (4.7%) 6 (1.3%)	0.091
Oocytes retrieved	16.4 (12.03)	15 (10.51)	0.545^*^
MII oocytes	13.1 (10.22)	11.7 (8.61)	0.364^*^
Oocyte maturity rate	78.1 (17.27)	77.4 (18.11)	0.719
Embryo cryopreservation No Yes	111 (90.2%) 12 (9.8%)	410 (90.9%) 41 (9.1%)	0.861
Sperm origin Donor Partner	11 (91.7%) 1 (8.3%)	31 (75.6%) 10 (24.4%)	0.421

Values are presented as mean (standard deviation) or number (%). A *p* value of <0.05 was considered significant.

* The *p* value was calculated after log transformation for normal distribution.

GnRH, gonadotropin-releasing hormone; PPOS, progestin-primed ovarian stimulation; GT, gonadotropins; E2, estradiol; P, progesterone; hCG, human chorionic gonadotropin; MII, metaphase II.

A multivariate linear regression analysis (Table 3) showed a negative effect of high levels of basal FSH and low levels of AFC on the total number of retrieved oocytes and on the number of MII oocytes, while there was no comparable correlation between these variables and the oocyte maturity rates. More advanced age of the participants showed a tendency for a negative correlation with the total number of retrieved oocytes and the number of MII oocytes. The protocol type (GnRH antagonist or PPOS) was not found to affect the various ovarian stimulation outcomes.

**Table 3:** Multivariate linear regression analysis for the number of retrieved oocytes, the number of MII oocytes, and the oocyte maturity rate.

Variable	Oocytes retrieved (n)		MII oocytes (n)		Maturity rate (%)	
	Standardized Coefficient (95% CI)	*p* value	Standardized Coefficient (95% CI)	*p* value	Standardized Coefficient (95% CI)	*p* value
Age (y)	-0.071 (0.967, 1.753)	0.07	-0.084 (-0.025, 0)	0.051	-0.018 (-0.994, 0.686)	0.719
Basal FSH (mIU/mL)	-0.250 (-0.03, -0.016)	<0.001	-0.184 (-0.026, -0.009)	<0.001	0.069 (-0.178, 0.929)	0.183
AFC (n)	0.469 (0.019, 0.026)	<0.001	0.443 (0.018, 0.027)	<0.001	0.029 (-0.217, 0.381)	0.590
Protocol type	-0.043 (-0.1, 0.027)	0.256	-0.023 (-0.094, 0.053)	0.586	0.013 (-4.318, 5.667)	0.791

MII, metaphase II; FSH, follicle-stimulating hormone; AFC, antral follicle count, CI, confidence interval.

## DISCUSSION

The results of this study indicated a rapid and sustained increase in elective FP after the outbreak of the Covid-19 pandemic that significantly surpassed the pre-pandemic levels. The increase was mostly due to single women in their mid-thirties who chose to undergo FP. There was no significant difference in FP outcomes before or after the pandemic.

A few other evaluated trends in infertility and ART service utilization rates during the pandemic. Zhou *et al*. (2021) found incremental increases in infertility and ART service utilization rates before the pandemic, sharp decreases during suspension of the services, and sharp recoveries after renewal of the services that were sustained throughout the study until its closure. Requena *et al*. (2020) observed a pronounced drop in all ART activity during the first outbreak of the Covid-19 pandemic, except for a gradual increase in urgent FP procedures. A higher volume of urgent FP cycles during the outbreak was also demonstrated by Trawick *et al*. (2022). In contrast, Roux *et al*. (2022) observed a decrease in medical FP activity during the lockdown, and a partial restoration of oocyte freezing activity after the lockdown compared with the pre-pandemic figures. To the best of our knowledge, we are the first to study the effect of the Covid-19 pandemic on elective FP rates to date.

There are several possible explanations for the significant increase in elective FP rates at the end of the lockdown. Numerous studies have suggested that approximately one-third of women have altered their childbearing or fertility treatment plans because of the Covid-19 pandemic (Zhu *et al*., 2020; Lindberg *et al*., 2020; 2021; Naya *et al*., 2021). These reported changes are predominantly characterized by a deferral of fertility most commonly associated with fear of pregnancy and childbirth, concerns about a non-optimal childrearing environment, and financial considerations associated with unstable employment conditions. Elective FP in the setting of deferral of family planning might be a motivation for pursuing oocyte cryopreservation. Huttler *et al*. (2022) evaluated the impact of the Covid-19 pandemic on attitudes toward planned oocyte cryopreservation. Those authors found that 15.2% of the 1000 responders believed that the pandemic influenced their likelihood of considering oocyte cryopreservation. Of those responders, 52.6% reported an increased likelihood and 47.3% reported a decreased likelihood of considering oocyte cryopreservation. Increased time working remotely because of the pandemic and fears from contact with Covid-19-positive individuals at the workplace were associated with a higher overall increased likelihood of considering elective oocyte cryopreservation. The authors assumed that these two parameters may limit interpersonal interactions and potential identification of a partner. Working from home also provided greater flexibility to attend appointments for undergoing FP, and the reduction in expenses due to lockdown restrictions led to savings that could be used for other purposes, such as FP. Huttler’s group (Huttler *et al*. 2022) also reported that nulliparity was significantly associated with consideration of oocyte cryopreservation, in line with our result of the increase in elective FP rate being due to the numbers of single women. This demographic predictor might reflect the evolution of the population seeking out FP services over time. However, the finding that the age of the patients (around 35 years) did not differ between our two study groups is interesting, especially since 35 is the threshold for a negative effect on fertility.

The PPOS protocol was used only in women who underwent elective FP after the Covid-19 outbreak. Progestin for pituitary suppression during ovarian stimulation is an equivalent alternative to the GnRH antagonist protocol in women undergoing FP (Ata *et al*., 2021; Guan *et al*., 2021). This protocol has gained considerable popularity, and we only recently started to use it routinely. The PPOS protocol makes FP more cost-effective for the women, which is an important consideration given that the significant costs associated with the procedure pose a major barrier to pursuing FP (Anderson *et al*., 2020), whereupon anything that lowers the price will make the process accessible to more women.

There was no difference in ovarian stimulation outcomes before and after the initial outbreak of the Covid-19 pandemic. Others have demonstrated that Covid-19 infection did not affect ovarian reserve (Kolanska *et al*., 2021; Madendag *et al*., 2022) or IVF outcomes (Bentov *et al*., 2021; Banker *et al*., 2022). It was assumed that the virus affected various organs through the ACE2 receptors and, although the ACE2 receptors are present in ovaries (Rajput *et al*., 2021), the presence of Covid-19 viral RNA in the oocytes or follicular fluid of infected women was not observed (Barragan *et al*., 2021; Boudry *et al*., 2022). Trawick *et al*. (2022) compared the FP outcomes among women who underwent urgent medical FP before and after pandemic and, similar to our results, found no differences between the groups. However, Trawick *et al*. (2022) reported that significantly more women pursued embryo cryopreservation rather than oocyte cryopreservation in 2020 (after the outbreak of the pandemic) compared with 2019 (before the outbreak) despite similar rates of partnership in both cohorts. Those authors hypothesized that the decision to cryopreserve embryos in lieu of oocytes may reflect a shift in reproductive decision-making during the pandemic. Their study included women with cancer who underwent urgent medical FP, while our study included mostly single healthy women who underwent elective FP who may have preferred freezing unfertilized eggs.

The present study has several limitations. Firstly, its retrospective design limits our ability to obtain more details about the correlation between the Covid-19 pandemic and the decision-making process with regard to FP. A prospective study using standardized questionnaires or interviews for eliciting reasons for considering FP in relation to the pandemic is recommended. Further, the lack of information on either the presence of Covid-19 infection or the status of vaccination precludes the ability to assess the impact of those parameters on both the women’s decision to undergo FP and on the outcomes of the process. Secondly, the study was conducted in a single institution located in the center of Tel Aviv, which is the most liberal area in Israel, a factor that may influence the women’s characterizations and preferences.

## CONCLUSION

We believe this to be the first study to demonstrate a rapid and sustained increase in elective FP utilization after the first outbreak of the Covid-19 pandemic. Additional studies are necessary to fully elucidate the impact of the pandemic on reproductive decision-making, particularly with regard to elective FP. There was no evidence of any adverse impact on ovarian stimulation outcomes associated with the pandemic. Future larger studies with longer follow-up will be needed to validate our observations.

## References

[r1] Alteri A, Viganò P, Maizar AA, Jovine L, Giacomini E, Rubino P. (2018). Revisiting embryo assisted hatching approaches: a systematic
review of the current protocols. J Assist Reprod Genet.

[r2] Anderson RA, Davies MC, Lavery SA, Royal College of Obstetricians and Gynaecologists (2020). Elective Egg Freezing for Non-Medical Reasons: Scientific Impact
Paper No. 63. BJOG.

[r3] ASRM - American Society for Reproductive Medicine (2020). Patient management and clinical recommendations during the coronavirus
(Covid-19) pandemic.

[r4] Ata B, Capuzzo M, Turkgeldi E, Yildiz S, La Marca A. (2021). Progestins for pituitary suppression during ovarian stimulation
for ART: a comprehensive and systematic review including
meta-analyses. Hum Reprod Update.

[r5] Banker M, Arora P, Banker J, Shah A, Gupta R, Shah S. (2022). Impact of COVID-19 Pandemic on Clinical and Embryological
Outcomes of Assisted Reproductive Techniques. J Hum Reprod Sci.

[r6] Barragan M, Guillén JJ, Martin-Palomino N, Rodriguez A, Vassena R. (2021). Undetectable viral RNA in oocytes from SARS-CoV-2 positive
women. Hum Reprod.

[r7] Bentov Y, Beharier O, Moav-Zafrir A, Kabessa M, Godin M, Greenfield CS, Ketzinel-Gilad M, Ash Broder E, Holzer HEG, Wolf D, Oiknine-Djian E, Barghouti I, Goldman-Wohl D, Yagel S, Walfisch A, Hersko Klement A. (2021). Ovarian follicular function is not altered by SARS-CoV-2
infection or BNT162b2 mRNA COVID-19 vaccination. Hum Reprod.

[r8] Boudry L, Essahib W, Mateizel I, Van de Velde H, De Geyter D, Piérard D, Waelput W, Uvin V, Tournaye H, De Vos M, De Brucker M. (2022). Undetectable viral RNA in follicular fluid, cumulus cells, and
endometrial tissue samples in SARS-CoV-2-positive women. Fertil Steril.

[r9] Cobo A, García-Velasco JA, Coello A, Domingo J, Pellicer A, Remohí J. (2016). Oocyte vitrification as an efficient option for elective
fertility preservation. Fertil Steril.

[r10] Cobo A, Garcia-Velasco JA, Domingo J, Remohí J, Pellicer A. (2013). Is vitrification of oocytes useful for fertility preservation for
age-related fertility decline and in cancer patients?. Fertil Steril.

[r11] Dunson DB, Baird DD, Colombo B. (2004). Increased infertility with age in men and women. Obstet Gynecol.

[r12] ESHRE - European Society of Human Reproduction and
Embryology (2020). Assisted Reproduction and Covid-19.

[r13] Faddy MJ, Gosden RG, Gougeon A, Richardson SJ, Nelson JF. (1992). Accelerated disappearance of ovarian follicles in mid-life:
implications for forecasting menopause. Hum Reprod.

[r14] Guan S, Feng Y, Huang Y, Huang J. (2021). Progestin-Primed Ovarian Stimulation Protocol for Patients in
Assisted Reproductive Technology: A Meta-Analysis of Randomized Controlled
Trials. Front Endocrinol (Lausanne).

[r15] Hodes-Wertz B, Druckenmiller S, Smith M, Noyes N. (2013). What do reproductive-age women who undergo oocyte
cryopreservation think about the process as a means to preserve
fertility?. Fertil Steril.

[r16] Hook EB. (1981). Rates of chromosome abnormalities at different maternal
ages. Obstet Gynecol.

[r17] Huttler A, Koelper N, Mainigi M, Gracia C, Senapati S. (2022). Impact of the COVID-19 pandemic on the perception of planned
oocyte cryopreservation in the United States. F S Rep.

[r18] Kolanska K, Hours A, Jonquière L, Mathieu d’Argent E, Dabi Y, Dupont C, Touboul C, Antoine JM, Chabbert-Buffet N, Daraï E (2021). Mild COVID-19 infection does not alter the ovarian reserve in
women treated with ART. Reprod Biomed Online.

[r19] Lindberg LD, Mueller J, Kirstein M, VandeVusse A. (2021). The continuing impacts of the COVID-19 pandemic in the United States:
Findings from the 2021 Guttmacher Survey of reproductive health
experiences.

[r20] Lindberg LD, VandeVusse A, Mueller J, Kirstein M. (2020). Early impacts of the COVID-19 pandemic: Findings the 2020 Guttmacher
Survey of reproductive health experiences.

[r21] Madendag IC, Madendag Y, Ozdemir AT. (2022). COVID-19 disease does not cause ovarian injury in women of
reproductive age: an observational before-and-after COVID-19
study. Reprod Biomed Online.

[r22] Mills M, Rindfuss RR, McDonald P, te Velde E, ESHRE Reproduction and Society Task Force (2011). Why do people postpone parenthood? Reasons and social policy
incentives. Hum Reprod Update.

[r23] Nasab S, Ulin L, Nkele C, Shah J, Abdallah ME, Sibai BM. (2020). Elective egg freezing: what is the vision of women around the
globe?. Future Sci OA.

[r24] Naya CH, Saxbe DE, Dunton GF. (2021). Early effects of the COVID-19 pandemic on fertility preferences
in the United States: an exploratory study. Fertil Steril.

[r25] Nybo Andersen AM, Wohlfahrt J, Christens P, Olsen J, Melbye M. (2000). Maternal age and fetal loss: population based register linkage
study. BMJ.

[r26] Rajput SK, Logsdon DM, Kile B, Engelhorn HJ, Goheen B, Khan S, Swain J, McCormick S, Schoolcraft WB, Yuan Y, Krisher RL. (2021). Human eggs, zygotes, and embryos express the receptor angiotensin
1-converting enzyme 2 and transmembrane serine protease 2 protein necessary
for severe acute respiratory syndrome coronavirus 2
infection. F S Sci.

[r27] Requena A, Cruz M, Vergara V, Prados N, Galliano D, Pellicer A. (2020). A picture of the covid-19 impact on IVIRMA fertility treatment
clinics in Spain and Italy. Reprod Biomed Online.

[r28] Rossman H, Keshet A, Shilo S, Gavrieli A, Bauman T, Cohen O, Shelly E, Balicer R, Geiger B, Dor Y, Segal E. (2020). A framework for identifying regional outbreak and spread of
COVID-19 from one-minute population-wide surveys. Nat Med.

[r29] Roux C, Pirello O, Clairaz P, Poirot C, GRECOT COVID GROUP. (2022). Impact of the COVID-19 pandemic on fertility preservation
activities in France: A survey by the Groupe de Recherche et d’Etude sur la
Conservation Ovarienne et Testiculaire (GRECOT; group for research and
studies on ovarian and testicular preservation). J Gynecol Obstet Hum Reprod.

[r30] Schmidt L, Sobotka T, Bentzen JG, Nyboe Andersen A, ESHRE Reproduction and Society Task Force (2012). Demographic and medical consequences of the postponement of
parenthood. Hum Reprod Update.

[r31] Sozou PD, Hartshorne GM. (2012). Time to pregnancy: a computational method for using the duration
of non-conception for predicting conception. PLoS One.

[r32] Trawick E, Babayev E, Potapragada N, Elvikis J, Smith K, Goldman KN. (2022). Fertility Preservation During the COVID-19 Pandemic: Modified But
Uncompromised. Womens Health Rep (New Rochelle).

[r33] WHO - World Health Organization (2020). Coronavirus disease (COVID-19) pandemic.

[r34] Zhou B, Joudeh A, Desai MJ, Kwan B, Nalawade V, Whitcomb BW, Su HI. (2021). Trends in Infertility Care Among Commercially Insured US Women
During the COVID-19 Pandemic. JAMA Netw Open.

[r35] Zhu C, Wu J, Liang Y, Yan L, He C, Chen L, Zhang J. (2020). Fertility intentions among couples in Shanghai under COVID-19: A
cross-sectional study. Int J Gynaecol Obstet.

